# Clinical Lecture in the Medical Ward of the Illinois General Hospital

**Published:** 1851-11

**Authors:** N. S. Davis

**Affiliations:** One of the Physicians to the Hospital


ARTICLE VII.

*Clinical Lecture in the Medical Ward of the Illinois General Hospital.* By N. S. Davis, M. D., one of the Physicians to the Hospital.

October 13th, 1851.—*Gentlemen:* The case before you, and the one to which I shall chiefly direct your attention this morning, is somewhat complicated and possessed of much practical interest. The patient is a female, aged about 24 years, unmarried, a native of Ireland, and before being afflicted with the disease was employed as a servant girl. You see in her countenance an expression of sadness or dejection, and in her arm, here, evidences of great emaciation, there being little left beside the bones, muscular fibres, and skin. Her pulse is 80 per minute, small and easily compressed; her tongue smooth, red, and moist; gums swollen, dark red or purple and spongy; skin dry and harsh; abdomen distended and somewhat tympanitic; bowels habitually very costive; thirst excessive, and appetite craving with painful and imperfect digestion. The urine, a specimen of which is contained in this vial, is pale or limpid like water, and very copious, amounting to about two gallons daily. Its specific gravity, as ascertained by Mr. Johnson, at present *Interne* of the Hospital, is 1030, that of healthy urine averages about 1020. According to the rule adopted by Dr. Christison for ascertaining the quantity of solid matter in 1000 parts of urine, the excess of specific gravity over 1000, is to be multiplied by 2.33, which would make this specimen contain 69,90, a considerable excess over the average of health. It also possesses a sweetish taste indicating the presence of sugar. To determine this important point with more certainty, however, we must resort to other tests. If I place a drachm or two in this test-tube, add to it a small quantity of the Solution of Caustic Potash contained in this bottle you see no immediate change, but on holding it in the flame of a spirit lamp until it is heated to the boiling point you see it rapidly assuming a deep orange yellow or redish color. This is called Moore's test, but it is not as reliable for detecting sugar as that devised by Tommer, which I will now apply. I place another drachm of the urine in this clean test-tube and add enough of the saturated Solution of Sulphate of Copper to give it a slight blueish color. I now add a Solution of Caustic Potash which throws down a copious green precipitate of ammoniate of copper, which by continuing to add the alkaline solution in excess, is re-dissolved leaving as you now see, a clear green solution. If I now hold this in the flame of the spirit lamp until the Ammonia is driven off the sugar in the urine is decomposed, part of its Oxygen unites with the Copper forming an insoluble red Oxide, the presence of which is indicated by the rapid change from the green to a very distinct red, as you see taking place here. The changes here detailed and exhibited to you are a very certain indication of the presence of sugar in the spicimen of urine examined.

And the results in this case enable us at once to arrive at the certain and important conclusion that the patient, just now before us is laboring under an aggravated degree of Diabetes Mellitus. She was admitted to the Hospital only three days since, and her history, as nearly as can be ascertained, is briefly as follows, viz: Eighteen months since while engaged in washing windows in one of the Hotels in our city, the usual period of menstural flow arrived, commenced in its usual regular and natural manner, but the patient continuing to work in the wet and cold it was entirely suppressed before the end of the first 24 hours, and has never returned since.

This was the beginning of ill health, she having previously enjoyed as good health and as regular a flow of the menstural fluid as could be desired. Sickness, however, soon followed the suppression and a Physician was called on who prescribed cathartics, alteratives, and emmenagogues, but with no other effect than the palliation of symptoms. During the first six months she had one or two attacks of fever, the last of which left her much debilitated and with a dry harrasing cough. The latter continued with gradually increasing emaciation until about six months since, when the cough gradually diminished and has now entirely disappeared. The emaciation, however, has continued to increase; she has become more and more anemic, and her vital energies more impaired until she presents the appearances and symptoms already detailed. The most careful examination, aided by Auscultation and Percussion, does not enable us to detect any tubercular deposits in the lungs or enlargements of any of the abdominal or pelvic vicersa.

It is now impossible to ascertain at what period in the progress of this case the Diabetic affection commenced, or how far it has operated to prevent the return of the Menstrual secretion. It is most probable that the sudden suppression was first followed by those congestive and inflammatory attacks which ordinarily occur in such cases. These were combatted and to a great extent, at least, removed by medical treatment, but the Menstrual function not being restored, the functions of the stomach and lungs next became permanently impaired, giving rise to costiveness, indigestion, and cough; at first directly threatening the development of tubercles and consumption, but the indigestion assuming that peculiar form or state, which, Prout and others suppose, gives rise to the conversion of the Carbonaceous portions of food into sugar, and its consequent introduction into the circulation in such quantities that it cannot be further assimilated or appropriated for nourishing the tissues, it soon stimulated the kidneys into excessive action, thereby commencing the diabetic affection which is now so plainly and fully developed. As a consequence of this protracted chain of morbid action, we have now great emaciation; an impoverished and anemic condition of the blood ; impairment of the solid textures indicated by the purple and spongy gums ; impairment of nervous energy both as regards the nerves of organic and animal life, and consequent loss of the peristaltic muscular actions producing obstinate costiveness and flatulency ; while Menstrual suppression still continues and the food which is taken in large quantities is converted into sugar only to be eliminated in the copious renal secretion. Such is the most rational pathological view of the past history and present condition of the case before us.

And what are the prospects and curative indications which it suggests?

The Prognosis in all cases of Diabetes Mellitus is unfavorable. The disease seems to have its origin in the defective or altered state of the digestive and assimilative functions, the precise causes and conditions of which are yet involved in obscurity, and hence we are without a completely rational and successful mode of treatment. The special Prognosis in the case before us is still more unfavorable from its origin and uterine complication. The remarks already made, however, readily suggest several well defined and important indications for treatment.

The first is, to cut off the supply of materials most readily converted into sugar, by a proper regulation of diet. Such materials are made up of the Carbonaceous class of aliments, and especially that variety chiefly composed of starch, as the farinaceous vegetables and tuberous roots. These, together with saccharine matters, should be carefully excluded, and the patient confined to an animal diet as exclusively as is consistent or compatible with the continuance of healthy digestion. A diet exclusively of muscular flesh is indicated by the disease, but there are very few patients who can maintain digestion any considerable length of time without a small supply of bread or other farinaceous article as a part of their food. The drinks should be bland and unstimulating, but may be allowed in liberal quantities without detriment and greatly to the comfort of the patients.

The second indication is to restrain the inordinate secretory action of the kidneys. For this purpose we have no other remedy equal in efficiency to Opium, given in moderate doses and repeated at regular intervals. Universal experience has given it the foremost rank as a paliative in the treatment of this disease. Its tendency to increase the constipation of the bowels is, however, a strong objection to its use, and sometimes renders its omission necessary. In many cases the Alkaline earths, especially the Oxides and Carbonates of Magnesia, are decidedly beneficial in lessening the urinary secretion, while they also tend to relax the bowels.

The third indication is to increase the tone of the digestive organs, improve the quantity and quality of the blood, and thereby arrest the emaciation and restore the healthy condition of the solid tissues. Aside from the proper regulation of diet, of which I have already spoken, the remedies best calculated to fulfill this indication are the different Salts of Iron and the bitter vegetable tonics. Of the preparations of Iron I have found none better generally that the Muriated Tincture in doses of from 10 to 20 drops three times a day. Within the last few days my attention has been called, by H. A. Johnson the intelligent Interne of these Wards, to an article which he had translated from a French Journal, detailing the use of a new remedy for Anæmia, called extract of Sanguinis. It is prepared by taking fresh beef's blood, allowing it to coagulate separating the serum through a filter and drying the clot by evaporation until it can be readily ieduced to a dry powder. Of this from ten to twenty grains are given three or four times a day. You will readily perceive that this, so called extract, is simply the red-corpuscles and fibrin of the blood, retaining all the Iron and a portion of the other saline constituents, in that form most nearly resembling their condition in the blood of the healthy living subject. Hence it contains the very constituents most needed to upbuild and improve such cases as the one before us.

The fourth, and last indication, is to restore the long suppressed Menstrual secretion. This, however, cannot be done unless the solids and fluids of the system are greatly improved and replenished first. The quantity and quality of the blood must first be so far restored as to furnish the materials for a healthy menstrual flow, or the whole list of direct and inindirect emmenagogues will be resorted to in vain. Indeed, in the present condition of the patient the very best remedies for fulfilling this indication, are indentical with those best calculated to fulfill the third as already alluded to.

With this brief exposition of the case, I shall direct the patient a diet of animal food consisting of Beef, Mutton, or Chicken either fresh or salted, and meat broth, with a very limited qnantity of wheaten bread. She will take of the Extract of Sanguinis, just described, 20 grs. three times a day; and a small powder of Aloes and Castile Soap or Carb. Magnesia as often as may be necessary to obviate costiveness. If the bowels become less constipated and the urine continues as copious as at present, half a grain of Opium may be added to each dose of the Extract after a few days.

The further progress of the case you will have abundant opportunities of observing during the term of Clinical Instruction which is now but just commenced.

